# Acoustic emission and fractal characteristics of red beds soft rock under water-force coupling

**DOI:** 10.1038/s41598-024-54814-2

**Published:** 2024-02-23

**Authors:** Lei Chen, Taiyu Liu, Baoxin Jia, Jupeng Tang, Jiashun Liu

**Affiliations:** 1 School of Civil Engineering, Liaoning Technical University, FuxinLiaoning Province, 123000 China; 2 Key Laboratory of Disaster Management and Ecological Restoration in Resource-Depleted Mining Areas of Liaoning Province, Fuxin, 123000 Liaoning Province China

**Keywords:** Tunnel engineering, Red beds soft rock, Uniaxial graded loading, AE, SEM, Fractal characteristics, Engineering, Civil engineering

## Abstract

Groundwater has a significant influence on the mechanical properties of surrounding rock. Aiming at the large deformation of surrounding rock of red layer soft rock tunnel affected by groundwater, the uniaxial graded loading tests were carried out on red beds soft rock with different water content. The failure process of the specimen was monitored by acoustic emission (AE) and the crack evolution law was analyzed, and the scanning electron microscopy (SEM) was used to compare the microstructure of the specimens before and after immersion. Combined with fractal theory, the monofractal and multifractal characteristics of AE ringing count during the loading process of red beds soft rock were analyzed. The results show that, with the gradual increase of water content, the AE ringing count before the yield stage gradually decreased, and the corresponding cumulative ringing count at the same time gradually decreased, and the decrease was large in the early stage of immersion, and decreased in the later stage. The cumulative ringing curve gradually slowed down, the internal crack appeared earlier, the cumulative ringing curve stepped significantly, the AE signal amplitude gradually weakened, and the bandwidth of each frequency band gradually decreased. The failure of red beds soft rock with different water content is dominated by shear crack, and with the gradual increase of water content, the proportion of shear crack increases gradually, and the AE *b* value decreases gradually. With the gradual increase of the relative peak strength, the correlation dimension *D* of red beds soft rock with different water content increases first and then decreases. At 80% of the relative peak strength, the correlation dimension *D* reaches its maximum value and then drops sharply until it is maintained at a relatively low level, and the correlation dimension *D* gradually decreases with the water content. The fitting correlation coefficients of different water content (ln*C*(*r*), ln*r*) are all above 0.9, indicating that the AE ringing count of water-bearing red beds soft rock has fractal characteristics, and the higher the correlation coefficient, the higher the self-similarity of AE ringing count sequence. As the weight *q* gradually increases, the generalized fractal dimension *D*(*q*) gradually decreases. When *q* ≠ 0, under the condition of the same *q*, *D*(*q*) presents a trend of first increasing and then decreasing. The multifractal characteristics of AE ringing count of red beds soft rock with different water content is inverted ‘U’ shape. From the natural state to immerse 1 d, the ∆*α* gradually increases, and from 1 to 7 d, the ∆*α* gradually decreases, where Δ*α* = *α*_max_ − *α*_min_ represents the spectral width of the multifractal spectrum. When saturation is not reached, ∆*f* < 0 indicates that the number of cracks in the specimen is small, when saturation is reached, ∆*f* > 0 indicates that a large number of cracks are generated inside the specimen and macro cracks are formed, where Δ*f* = *f* (*α*_max_) − *f* (*α*_min_) represents the frequency relationship between different signals of different sizes. This research can provide a reliable theoretical basis for the construction and maintenance of large deformation of water-rich soft rock tunnel excavation, and have certain engineering significance.

## Introduction

Rock is a complex geological body formed by long geological structure movement, and its interior often contains different degrees of original defects. Under the loading condition, the original defects inside the rock are compressed and closed, new cracks develop and penetrate, and finally form macroscopic cracks and fail to be stable^1,2^. In this process, the generation of micro-cracks will induce acoustic signals of different frequencies, that is, the AE of the rock. The signal can directly describe the whole process of rock progressive failure, which is a common non-destructive monitoring technology used to monitor rock failure in the world, and has very important significance for the stability monitoring and disaster prediction of underground engineering surrounding rock.

In recent years, AE (Acoustic Emission) has been widely used in the field of rock mechanics. Lu et al.^3^ used numerical simulation to study the triaxial compression characteristics and acoustic emission evolution characteristics of the backfill-rock composite structure in view of its deformation and failure. Fan et al.^4^ conducted a series of shear tests on different types of stratified shales, monitored the failure process of the specimens with AE, and analyzed the shear failure mechanism of different types of stratified shales. Akdag et al.^5^ conducted a series of triaxial compression tests on the granite in view of the rockburst problem faced in the process of deep resource mining, and used a newly developed acoustic emission technology to study the post-peak energy evolution.In order to study the failure behavior of cracked rock, Liu et al.^6^ conducted quasi-static compression tests on different types of prefabricated cracked rock, and analyzed the evolution mechanism of cracks with the help of AE. In order to study the precursor characteristics and principal stress direction of rock instability failure, Dong et al.^7^ used acoustic emission technology to monitor the deformation and failure process of rock and revealed the relationship between AE wave velocity and rock anisotropy. Dou et al.^8^ conducted uniaxial compression tests on sandstone specimens with different prefabricated crack inclination angles, and used AE to monitor the internal crack propagation of the specimens. He et al.^9^ studied the precursor characteristics of rock failure using AE and proposed a warning method for the ‘quiet period’ before failure. Xue et al.^10^ conducted triaxial compression and seepage tests on coal specimens under different gas pressures, and used AE to monitor the failure process under different test conditions. Song et al.^11^ conducted a uniaxial cyclic loading and unloading test on brittle coal specimens, used AE to monitor the crack distribution inside the specimens, and analyzed the crack evolution mechanism under cyclic loading. Dong et al.^12^ studied the failure mechanism of granite through biaxial compression test and AE test, and analyzed the influence of different intermediate principal stresses on the deformation and failure process. Zhang et al.^13^ studied the physical and mechanical properties and cracking mechanism of ananite rock through uniaxial compression test, AE test, NMR (Nuclear Magnetic Resonance) test and SEM (Scanning Electron Microscopy) test. Miao et al.^14^ in order to study the damage and rupture mechanism of rocks under slight disturbance and high static state, used AE test to monitor the damage process of micro-disturbed rocks. Chu et al.^15^ studied the tensile properties and failure modes of bedding joint rocks through the Brazilian splitting test, and analyzed the failure mechanism and crack propagation law of bedding joint rocks by using AE, DIC (Digital Image Correlation) and SEM tests. Xue et al.^16^ studied the crack evolution characteristics of granite through triaxial compression and AE tests, and analyzed the spatial correlation of AE events in the specimens by using cube clustering model. Zhang et al.^17^ analyzed the unloading failure characteristics of marble through the triaxial unloading test, tracked and monitored the crack development during the failure process with the aid of AE, and analyzed the evolution law of AE *b* value based on the test results.

On the other hand, fractal theory has been widely used in the process of rock deformation and failure. Xiao et al.^18^ studied the deformation and failure mechanism of coal and rock caused by gas adsorption through laboratory tests, and analyzed the relationship between adsorption damage and fractal dimension combined with fractal theory. Huang et al.^19^ studied the evolution law and fractal characteristics of AE parameters during deformation and failure of sandstone under different confining pressures through triaxial compression AE tests. Wang et al.^20^ studied the pore characteristics and mechanical properties of white sandstone through multi-field coupling tests, quantified the crack evolution characteristics, and analyzed the relationship between fractal dimension and damage. Gao et al.^21^ conducted uniaxial compression tests on five kinds of rocks, and studied the dynamic failure characteristics of five kinds of rocks based on fractal theory. Ma et al.^22^ studied the inelastic characteristics of reservoir rocks through acoustic wave measurement tests, and established a two-hole fractal elastic model with self-similar characteristics combined with fractal theory. Biswas et al.^23^ studied the rocks in eastern India through shear tests, and analyzed the fractal characteristics of the roughness of the shear zone of the rocks in this region by fractal theory. Liu et al.^24^ established a multivariate analysis system for cracked rock mass structural planes based on manifold learning and fractal theory, and proposed the analysis process of monofractal and multifractal. Zhang et al.^25^ conducted experimental studies on 13 typical coal measure sedimentary rocks by using NMR, and realized the application of multifractal in fluid mobility of coal measure sedimentary rocks based on fractal theory. Xi et al.^26^ used FESEM (Field Emission Scanning Electron Microscopy) and NMR to analyze the formation and propagation of cracks in granite after cold and heat treatment, and established a theoretical model of rock damage evolution based on fractal theory. Gogus et al.^27^ analyzed the damage of triaxial rock before failure by conducting uniaxial compression tests on ophiase, lignite and marble, and quantitatively analyzed the damage process of rock based on fractal theory and SEM images. Sun et al.^28^ analyzed the relationship between AE events and stress of coal-rock by using fractal theory and Grassberger–Procaccia algorithm, and described the damage degree of coal-rock under multi-stage dynamic load. Zhang et al.^29^ studied the tensile failure characteristics of shale through the Brazilian splitting test and AE test, and analyzed the crack development characteristics during the failure process by using fractal theory.

In summary, the mechanism of AE monitoring rock deformation and failure has been studied in detail, but there are few studies on the monofractal and multifractal characteristics of AE during deformation and failure of water-force coupling red beds soft rock under uniaxial graded compression. In this paper, uniaxial graded loading tests are carried out on red beds soft rock with different water content, and the microstructure and failure process of the specimens before and after immersion are studied by SEM and AE. Based on fractal theory, the AE fractal characteristics of red beds soft rock in water-bearing are analyzed, and the failure mechanism of surrounding rock in large deformation tunnel under groundwater is revealed, which provides a reliable theoretical basis for excavation and support of underground engineering.

## Experimental introduction

**Figure 1:**
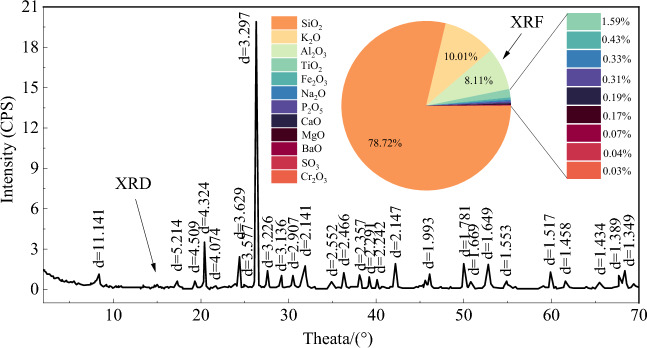
XRD and XRF test results of red beds soft rock.

The red beds soft rock used in this experiment was taken from a tunnel construction site under construction in Sichuan China. The rock block is transported to the indoor rock mechanics laboratory for cutting and polishing, and finally a cylindrical specimen with a height of 100 mm and a diameter of 50 mm, satisfying the ISRM (International Society for Rock Mechanics), was obtained. To minimize errors due to rock anisotropy, all specimens are taken from the same rock block. A grinding machine is used to polish the prepared rock specimen, so that the error of the specimen end face is within 0.02 mm as far as possible. TTR-III XRD (X_Ray Diffractometer) is used to detect the elemental composition and Bruker-S1-TITAN XRF (X_Ray Fluorescence) spectrometer is used to detect the chemical composition of red beds soft rock. And the results are shown in Fig. 1. Based on the test results, the chemical composition of the red beds soft rock includes SiO_2_ (78.72%), K_2_O (10.01%), Al_2_O_3_ (8.11%), TiO_2_ (1.59%), Fe_2_O_3_ (0.43%), Na_2_O (0.33%), P_2_O_5_ (0.31%), CaO (0.19%), MgO (0.17%), BaO (0.07%), SO_3_ (0.04%), Cr_2_O_3_ (0.03%).

**Figure 2:**
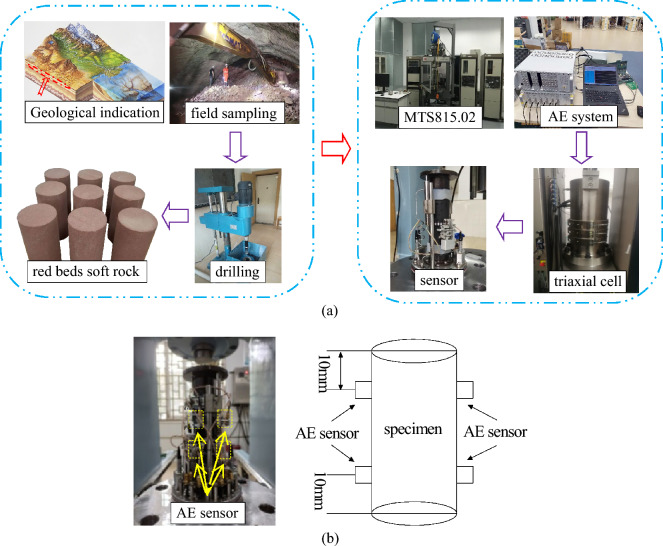
Red beds soft rock uniaxial graded loading experiment.

The uniaxial graded loading tests of red beds soft rock are carried out on MTS815.02 rock triaxial test system. As the red beds soft rock has low strength and relatively weak stability, the axial load is applied to the specimen by graded loading at a loading rate of 0.1 kN/min during uniaxial compression test. When the axial load reaches 10, 15, 20, 25, and 30 kN respectively, maintain a constant axial pressure for 60 s. Before loading, a small amount of Vaseline should be applied to both ends of the specimen to reduce the influence of uneven end surfaces on the test results. AE system adopts multi-channel PCI-II AE monitoring system produced by American Physical Acoustics Company. The system has high accuracy and fast sampling rate, and has been widely praised around the world. According to the test requirements, 4 AE probes are symmetrically arranged on the specimen surface, about 10 mm from the end height. Add a small amount of Vaseline between the specimen and AE probe to enhance their coupling, and fix them with transparent tape. The preamplifier gain is 40 dB, and the data acquisition frequency is 5 MHz, and the threshold is 110 mV, the noise is filtered through the system’s own filter, and the loading process is synchronized with the monitoring. Figure 2 shows the overall experimental process.

In order to study the influence of different water content on the mechanical properties of red beds soft rock, the specimens are immersed in water before the uniaxial graded compression experiment. The immersion time is 0 d, 1 d, 3 d and 7 d respectively. The water content of the specimens under different immersion time is tested, and the results are shown in Table 1. All rock specimens should be dried at 105℃ before immersion.

**Table 1:** Water content of red beds soft rock at different immersion time.

No	Time (d)	Before immersion (g)	After immersion (g)	Difference (g)
D01	0	455.75	475.41	19.66
D02		466.53	486.63	20.10
D03		473.77	494.22	20.45
D11	1	464.90	486.81	21.91
D12		425.93	445.10	19.17
D13		459.31	479.86	20.55
D31	3	464.90	490.84	25.94
D32		473.68	497.24	23.56
D33		486.88	511.47	24.58
D71	7	482.13	508.27	26.14
D72		468.66	496.07	27.41
D73		480.22	508.61	28.38

**Figure 3:**
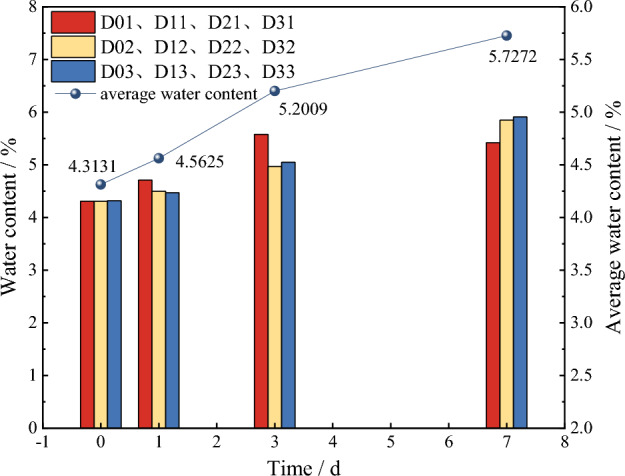
Relationship between water content and time of red beds soft rock.

According to Table 1, the water content of red beds soft rock at different immersion times is calculated, as shown in Fig. 3. In order to be closer to the actual engineering situation, the water content of the red beds soft rock under water in this paper refers to natural saturation. With the gradual extension of immersion time, the water content of the specimen increases gradually, but the increase rate decreases gradually. In the natural state, which refers to an immersion time of 0 days, the average water content of the specimen is 4.3131%. When the immersion time is 1 d, 3 d and 7 d, the water content is 4.5625%, 5.2009% and 5.7272% respectively, and the increase rate of water content per unit time is 0.2494%, 0.2128% and 0.1316%, respectively, where per unit time refers to every day. The water content increases with the extension of immersion time. However, the unit time water content increases by 0.1316% after being immersed for 7 days, which is less than 0.15%, and combined with actual engineering situation, the rock specimen tends to be saturated at this time.

## Analysis of experiment results

### Analysis of stress–time curve

**Figure 4:**
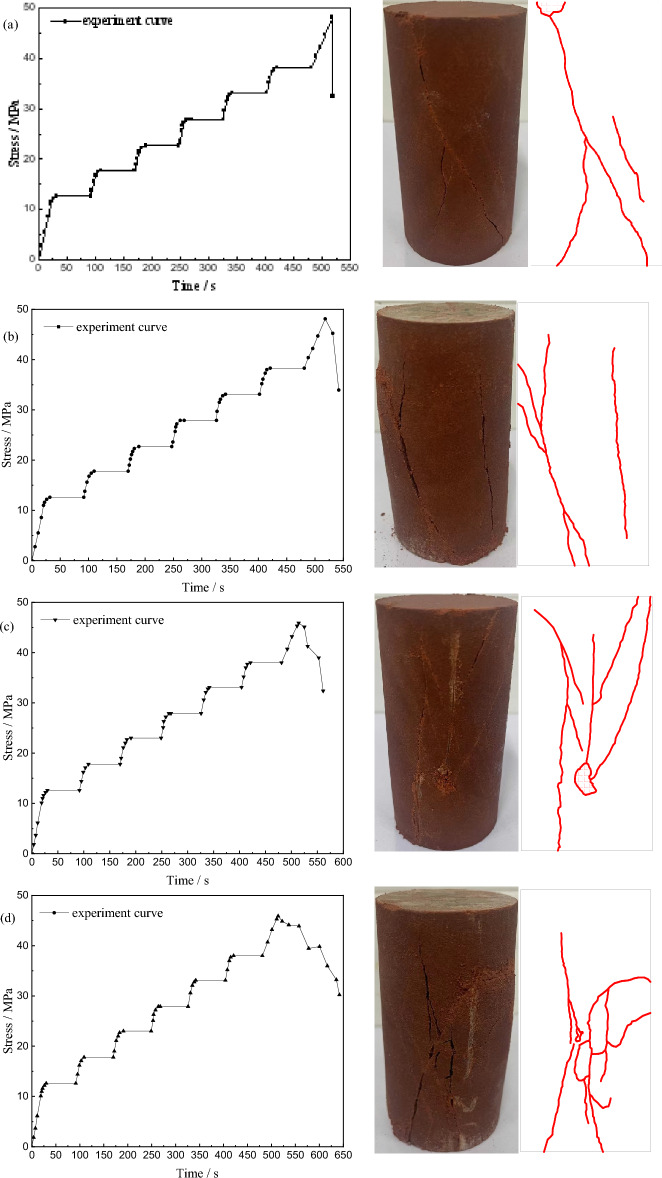
Stress–time curve and failure mode of red beds soft rock with different water content: (**a**) natural state (**b**) 1 d (**c**) 3 d (**d**) 7 d.

Figure 4 shows the stress–time curve of uniaxial graded loading experiment of red beds soft rock at different immersion times. The variation trend of stress–time curve under different conditions is basically the same. Before failure strength, the stress increases gradually with time, and when the failure strength is reached, the stress begins to drop. The difference is that with the extension of immersion time, the failure strength gradually decreases, and the post-peak curve gradually slows down. When the specimen is in a natural state, there is an oblique crack running through the surface, and two associated cracks appear in the lower part. The specimen as a whole showed shear brittle failure. With the extension of immersion time, the failure mode changes, the oblique cracks gradually disappear, and a number of complex and irregular cracks appear, and the failure mode gradually transitions to ductile.

**Figure 5:**
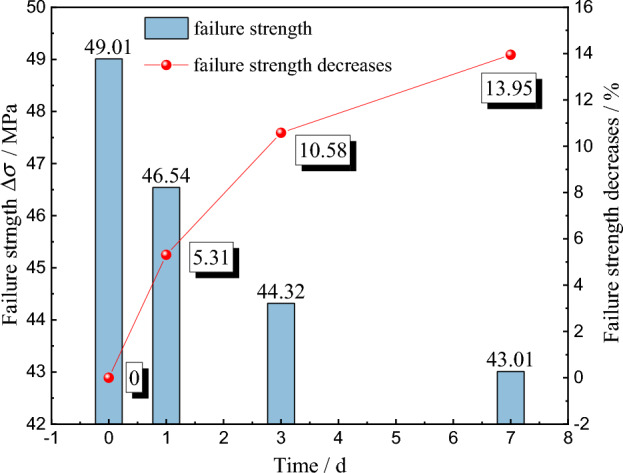
Relationship between failure strength and immersion time.

According to the uniaxial graded loading test results of red beds soft rock, the peak strength of specimens with different immersion time is obtained, as shown in Fig. 5. In the natural state, the peak strength of red beds soft rock is 49.01 MPa. After immersion for 1 d, 3 d and 7 d, the peak strength of the specimen came to 46.54 MPa, 44.32 MPa and 43.01 MPa, with decreases of 5.31%, 10.58% and 13.95%, respectively, indicating that water can significantly reduce the bearing capacity of rock materials. On the one hand, water enters the interior along the cracks on the rock surface, weakening the frictional bite between particles, thus reducing the cohesion. On the other hand, water percolates inside the rock, carrying away part of the free particles, reducing the bearing capacity of the internal skeleton structure.

### AE ringing characteristics analysis

**Figure 6:**
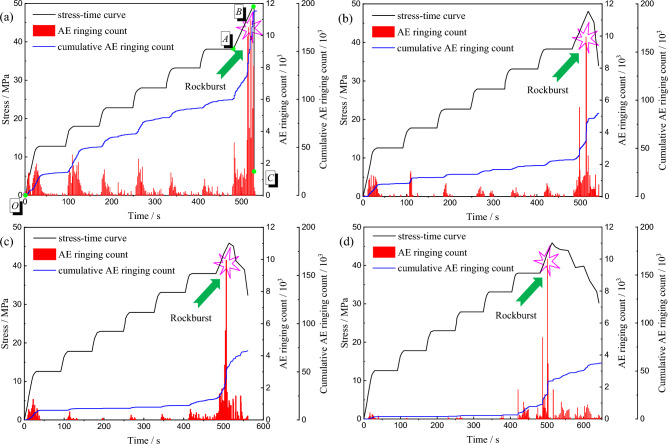
Red beds soft rock of stress-time-AE ringing count curve: (**a**) natural state (**b**) 1 d (**c**) 3 d (**d**) 7 d.

AE is widely used in rock mechanics, and ringing count is a general technique for AE evaluation. When the rock failure event hits the sensor, the electrical signal formed will be recorded as a ringing count once it exceeds the threshold^30^. Figure 6 shows the stress-time-AE ringing count curve of red beds soft rock with different immersion time. Taking the natural state specimen in Fig. 6a as an example, the stress–strain curve of red beds soft rock under uniaxial graded loading can be roughly divided into three stages, including the elastic stage *OA*, the plastic yield stage *AB* and the post-peak stage *BC*, where point *B* is the peak strength. The natural state specimens show relatively active ringing phenomenon and relative peak value in the early stage of each loading stage. As the stress gradually stabilizes, the ringing count plummets and fluctuates at a relatively low level, and the cumulative ringing curve gradually slows down. The reason is that the original microfissure inside the rock is gradually compacted. With the gradual increase of stress, the cumulative ringing count increases gradually, the curve begins to appear "steps", and the relative peak value of ringing count decreases gradually. When the stress reaches the failure strength, the cracks gradually expand and go through, and the AE activity increases substantially, which is manifested as a sharp increase in ringing count. After that, the specimen is destroyed in a very short time, accompanied by a violent sound, the stress dropped rapidly, the slope of the cumulative ringing count curve reached a peak, and the maximum ringing count is 9522. In the stage of graded loading, the average ringing count of red beds soft rock is 296, accounting for 3.1% of the maximum ringing count, and the cumulative ringing count is 74,025, accounting for 22.34% of the total cumulative ringing count.

Figure 6b–d show the stress-time-AE ringing count curves of red beds soft rock immersion in water for 1 d, 3 d and 7 d, respectively. Under different moisture content, the AE ringing curve changes significantly, especially after immersion for 7 d, the water content reaches 5.72%, and the AE ringing activity in the stage of graded loading is significantly reduced. Before the specimens entered the yield stage, the cumulative ringing count curve is basically horizontal. Compared with before immersion, the “step” is weakened, and the AE ringing activity after the peak gradually increased. Song et al.^31^ also reached a similar conclusion when conducting uniaxial compression tests on weakly consolidated rocks with different dry–wet cycles, indicating that water has a significant weakening effect on rocks.

**Figure 7:**
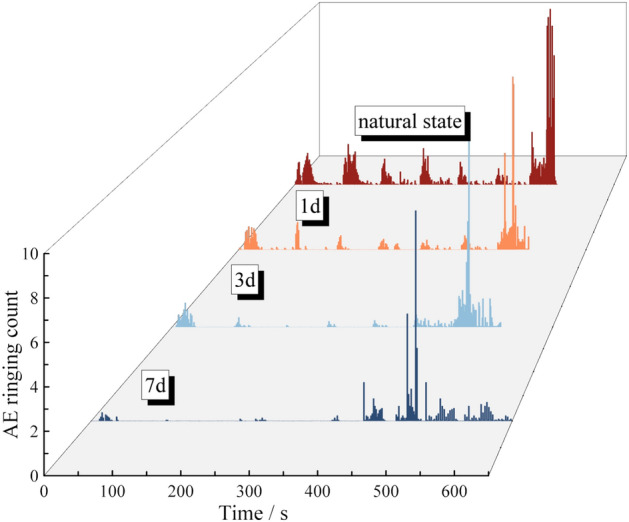
AE ringing comparison of soft rock in red beds and different water content.

**Figure 8:**
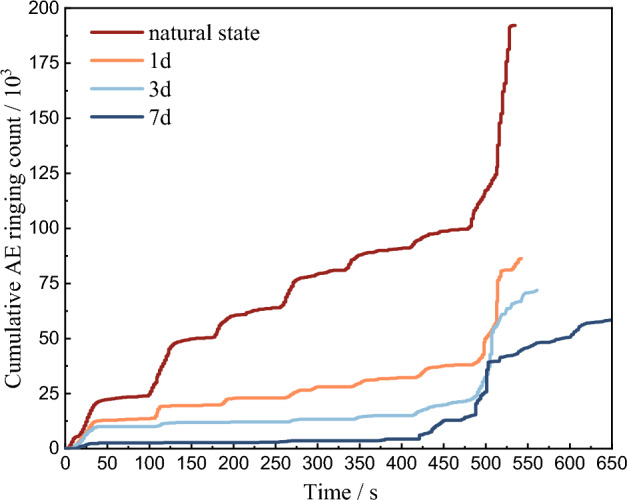
Cumulative ringing count and time curve of red beds soft rock and different water content.

In order to more intuitively compare AE ringing counts of red beds soft rock with different water content, AE ringing curves under four conditions are jointly drawn in Fig. 7. With the increase of water content, the AE ringing count before the yield stage decreases gradually. According to the curve of cumulative ringing count and time shown in Fig. 8, with the extension of immersion time, the corresponding cumulative ringing count at the same time gradually decreases, and the decrease is large in the early stage and gradually decreases in the later stage, which is basically consistent with the trend of rock water content with time, and the early stage refers to 0 d and 1 d, the later stage refers to 3 d and 7 d. After immersion for 1 d, 3 d and 7 d, the total cumulative ringing count is 256,781, 208,624 and 126,852, respectively, which is 77.49%, 60.96% and 38.28% of 331,356 in its natural state. In general, in the stage of graded loading, the relative peak value is small, and the cumulative ringing curve gradually slows down. In the yield stage, the longer the immersion time, the higher the water content, the earlier the internal crack appeared. The cumulative ringing curve has a more significant step with the increase of water content, and this significant step increase is the precursor of rock short imminent failure.

**Figure 9:**
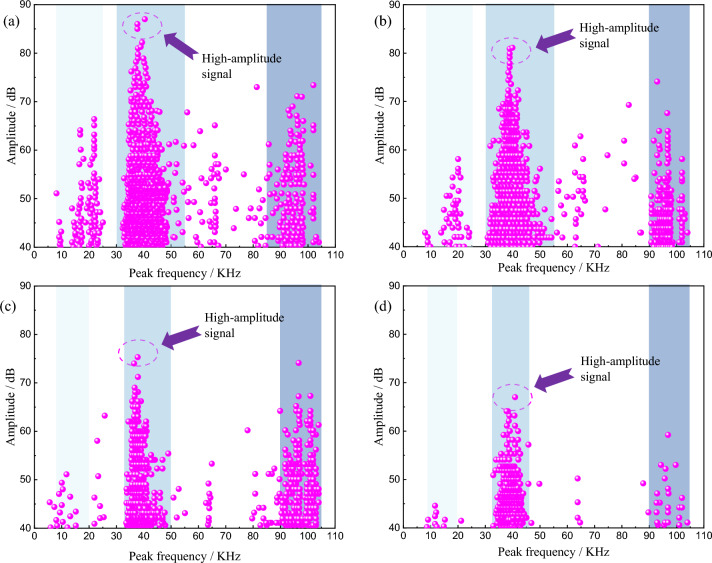
Relationship between AE amplitude and peak frequency of red beds soft rock with different water content: (**a**) natural state (**b**) 1 d (**c**) 3 d (**d**) 7 d.

According to the AE experiment, Matlab is used to process the experiment data, and the relationship between AE event amplitude and peak frequency with different water content is obtained^32^, as shown in Fig. 9. The peak frequency with different water content is roughly divided into three frequency bands: low frequency 10–25 kHz, medium frequency 30–55 kHz and high frequency 90–105 kHz. The AE high-amplitude signals all occur in the medium frequency band. With the gradual increase of water content, the amplitude of AE signal of red beds soft rock gradually decreases, and the bandwidth of each frequency band gradually decreases, and the signal of low frequency band decreases most obviously. When the specimen is immersed for 7 d, the signal of low frequency and high frequency band almost disappeared, and the AE amplitude signal is mainly composed of medium frequency band.

### Analysis of AE characteristic value of *R*_A_, *A*_F_ and *b*

**Figure 10:**
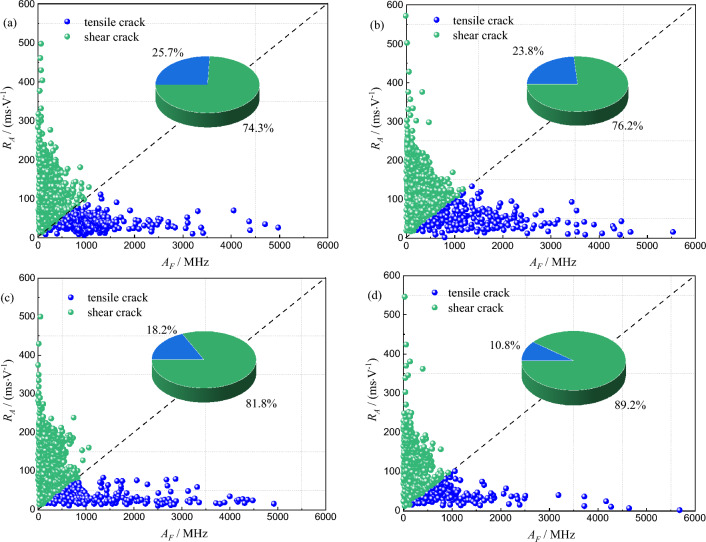
Distribution characteristics of tensile and shear cracks in red beds soft rock with different water content: (**a**) natural state (**b**) 1 d (**c**) 3 d (**d**) 7 d.

In the process of AE signal processing, there are two parameters that can characterize the type and proportion of cracks inside rocks, namely *R*_A_ and *A*_F_, which reflect the ascending angle and average frequency respectively. *R*_A_ is the ratio of the rise time of AE signal to the maximum amplitude of the corresponding signal wave, and *A*_F_ is the ratio of the ringing count of AE signal to the duration of the corresponding signal^33,34^. In general, AE signals with low *A*_F_ values and high *R*_A_ values indicate the generation or development of shear cracks. On the contrary, AE signals with high *A*_F_ values and low *R*_A_ values indicate the generation or development of tensile cracks. The distribution characteristics of shear and tensile cracks of red beds soft rock with different water content are shown in Fig. 10. The failure of red beds soft rock with different water content is mainly shear crack, and with the gradual increase of water content, the proportion of shear crack increases gradually, which are 74.3%, 76.2%, 81.8% and 89.2%, respectively. On the contrary, the proportion of tensile cracks decreased gradually, which are 25.7%, 23.8%, 18.2% and 10.8%, respectively. The gradual increase of shear cracks in beds soft rock is due to the micro-mechanism of various factors under the action of different water content states. The cracks in rock under the action of water are mainly shear. Under the action of external force, the rock compresses, and the pore water pressure existing in the internal crack provides a extrusion pressure to the tip of the original crack, causing the original crack to split, accompanied by the emergence of new cracks, the tip crack is fully developed, the degree of fragmentation is aggravated, and the more water in the rock, the more obvious the above situation.

**Figure 11:**
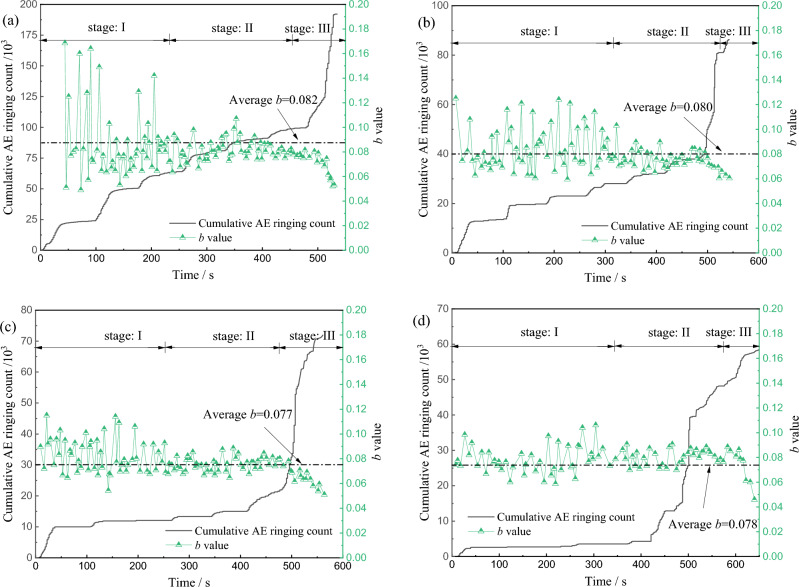
Evolution curve of AE *b* value of red beds soft rock with different water content: (**a**) natural state (**b**) 1 d (**c**) 3 d (**d**) 7 d.

Acoustic emission is the phenomenon of sound waves released by strain energy in the process of rock deformation and failure. AE is identified as a kind of microseismic activity. The change process from micro-crack initiation to macro-crack development during rock deformation and failure can be accurately reflected by the relative parameter *b* value of earthquake magnitude and frequency^35^. When *b* value decreases, it indicates that the proportion of small AE events decreases and the proportion of large AE events increases. When *b* value increases, it indicates that the proportion of small events increases and the proportion of large events decreases. The *b* value changes steadily and the range is small, indicating that the frequency of AE events is stable, that is, the development of cracks in the rock is a gradual and stable expansion. The steep drop of *b* value indicates that the crack development is severe and the rock is about to be destroyed. The evolution law of AE* b* value of red beds soft rock with different water content is shown in Fig. 11.

It can be seen from the figure that the *b* value always fluctuates during the whole loading process, and the change law of *b* value of soft rock in red beds with different water content is similar, which can be roughly divided into three stages, wave stage I, stable stage II and decay stage III.

In stage I, *b* value fluctuates up and down with the increase of load, the amplitude is large, and *b* value is relatively high. The main reason is that the rock is in the stage of microfissure compaction and linear elastic deformation at the initial loading stage, and the AE signal is generated by the original microfissure and pore structure compaction, which is a small event in response to the AE, so the fluctuation range of *b* is large. In stage II, with the continuous increase of load, the amplitude of the curve decreases, the proportion of acoustic emission events tends to balance, and the b value gradually enters a stable stage, reflecting that the cracks in the rock are steadily expanding. In stage III, as the load continues to increase, the *b* value begins to decline, and the proportion of large signals emitted by AE response increases, reflecting that the tiny cracks in the rock begin to merge and form larger cracks. When the peak strength is reached, the large cracks further expand to form macroscopic cracks, the rock is destroyed, and the *b* value is reduced to the lowest.

The comparison of different water content shows that the change of *b* value at the initial stage of loading is different. With the increase of water content, the fluctuation range of *b* value decreases gradually. In the natural state, the *b* value fluctuates in the range of 0.04–0.20, with an average value of 0.082, and when immersed for 7 days, the *b* value fluctuates in the range of 0.04–0.12, with an average value of 0.078. At the initial stage of loading, *b* value is greatly affected by water content. After immersion, water enters into the interior along the rock surface micro-cracks, which reduces the biting force between crack surfaces, and the interior of the specimen is full of water. As a result, AE signals weaken under axial loading, and *b* value decreases.

## Fractal theory and AE characteristic analysis

### AE monofractal characteristics of red beds soft rock with different water content

Fractal theory is widely used in rock mechanics and engineering. Based on fractal theory, Grasssberger proposed G-P algorithm to directly obtain correlation dimension *D* from time series^36^. Using this method, the AE parameter sequence during rock loading and failure process is taken as the research object, then each parameter sequence has a series set with a capacity of *n* corresponding to it:

1 $$ Y = \left\{ {y_{1} ,y_{2} , \ldots ,y_{n} } \right\}. $$

According to Eq. (1), a phase space of dimension *m* (*m* < *n*) can be obtained. First, *m* elements in Eq. (1) are taken as the first vector of the *m*-dimensional phase space:

2 $$ Y_{1} = \left\{ {y_{1} ,y_{2} , \ldots ,y_{m} } \right\}. $$

Then, obtain the second vector of the *m*-dimensional phase space:

3 $$ Y_{2} = \left\{ {y_{2} ,y_{3} , \ldots ,y_{m + 1} } \right\}. $$

By analogy, the *T* = *n*-*m* + 1 vector of *m*-dimensional phase space can be formed, and its corresponding correlation function can be obtained:

4 $$ C\left( r \right) = \frac{1}{T} \cdot \sum\limits_{i = 1}^{T} {\sum\limits_{j = 1}^{T} {H\left[ {r - \left| {x_{i} - x_{j} } \right|} \right]} } $$

where *H* is the Heaviside function, which can be expressed by the following equation:

5 $$ H\left( x \right) = \left\{ {\begin{array}{*{20}l} {0,} \hfill & {\left( {x < 0} \right)} \hfill \\ {1,} \hfill & {\left( {x \ge 0} \right)} \hfill \\ \end{array} } \right. $$

*r* is a known scale, and each *r* has a *C*(*r*) corresponding to it. In order to avoid discreteness in the value of *r*, it can be expressed by the following equation:

6 $$ r = \lambda r_{0} $$

where *λ* is the proportional coefficient, and *r*_0_ can be expressed as:

7 $$ r_{0} = \frac{1}{{T^{2} }} \cdot \sum\limits_{i = 1}^{T} {\sum\limits_{j = 1}^{T} {\left| {x_{i} - x_{j} } \right|} } $$

According to the above Eqs. (1)–(7), *n* coordinate points of (ln*r*, ln*C*(*r*)) can be obtained. Plot the obtained n points in log–log coordinates and fit the curve. If the fitting result is linear, it indicates that the AE sequence has monofractal characteristic within a known scale, and the slope of the fitting curve is the correlation dimension, which can be expressed by the following equation:

8 $$ D = \ln C\left( r \right)/\ln r. $$

**Figure 12:**
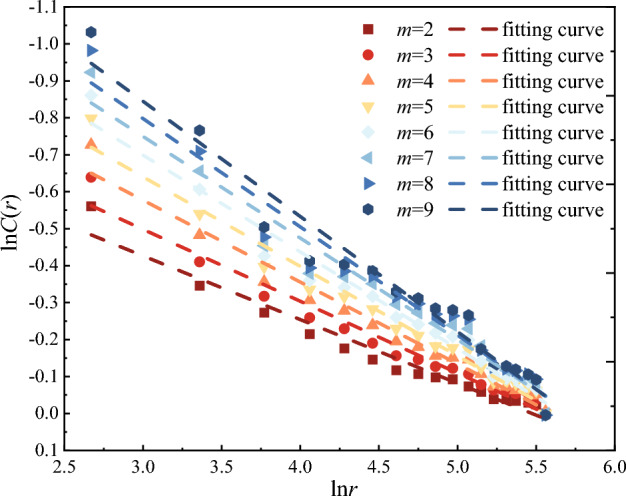
ln*C*(*r*) − ln*r* curves of different *m*-dimensional phase space.

**Figure 13:**
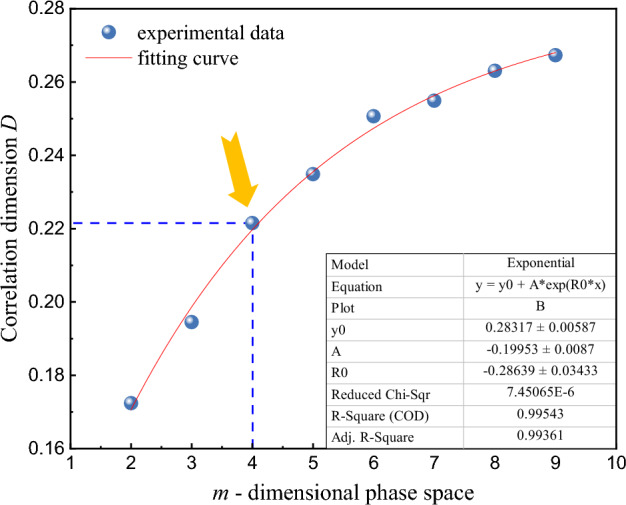
Relationship between correlation dimension *D* and *m*-dimensional phase space.

In order to clarify the influence of *m*-dimensional phase space on correlation dimension *D*, specimen D03 is taken as an example. With *r* unchanged, ln*C*(*r*)−ln*r* curves with different *m*-dimensional phase space are drawn in Fig. 12. With the gradual increase of *m*-dimensional phase space, the curve becomes steeper and the correlation dimension *D* gradually increases, but the increase is decreasing. It can be seen from Fig. 13, when *m* = 4, the increase of correlation dimension *D* starts to be less than 1.0%, and it is considered that the correlation dimension *D* is basically stable at this time, and the corresponding *m* = 4 can be used as the calculation parameter in this paper.

**Figure 14:**
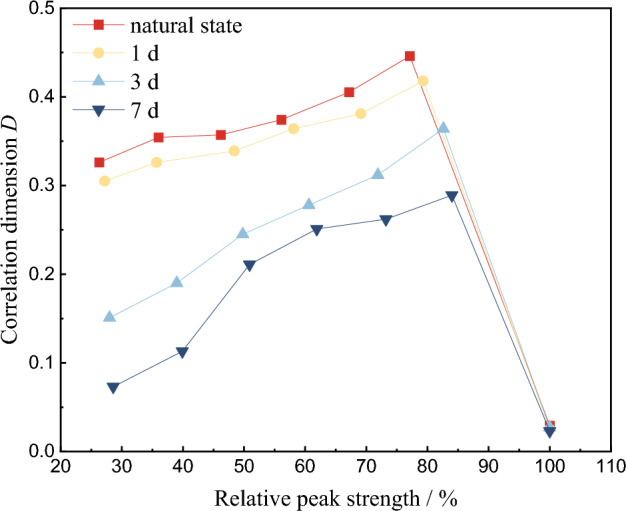
Relationship between relative peak intensities and correlation dimension *D*.

AE activities during the whole process of red beds soft rock failure are monitored. Using the ringing count as parameter, 100 AE ringing count data in each loading stage are calculated. The distribution law of correlation dimension *D* under different water content and stress levels is obtained, as shown in Fig. 14. With the gradual increase of relative peak strength, the correlation dimension *D* of rock specimens with different water content increases first and then decreases. The correlation dimension *D* reaches its maximum value at 80% of the relative peak strength, and then starts to drop sharply and maintains at a relatively low level *D* = 0.025–0.030. Near the relative peak strength of 80%, the correlation dimension *D* gradually decreases with the increase of water content. The reason is that the water immersion increases the water pressure between the joints and cracks inside the rock, and in this process, the water plays the role of strong infiltration, promoting cracks and reducing shear force, which effectively reduces the generation of AE signals. When the peak strength is reached, the correlation dimension *D* of different water content suddenly decreases, indicating that the AE signals generated by rock failure and instability are more and more disorderly.

**Figure 15:**
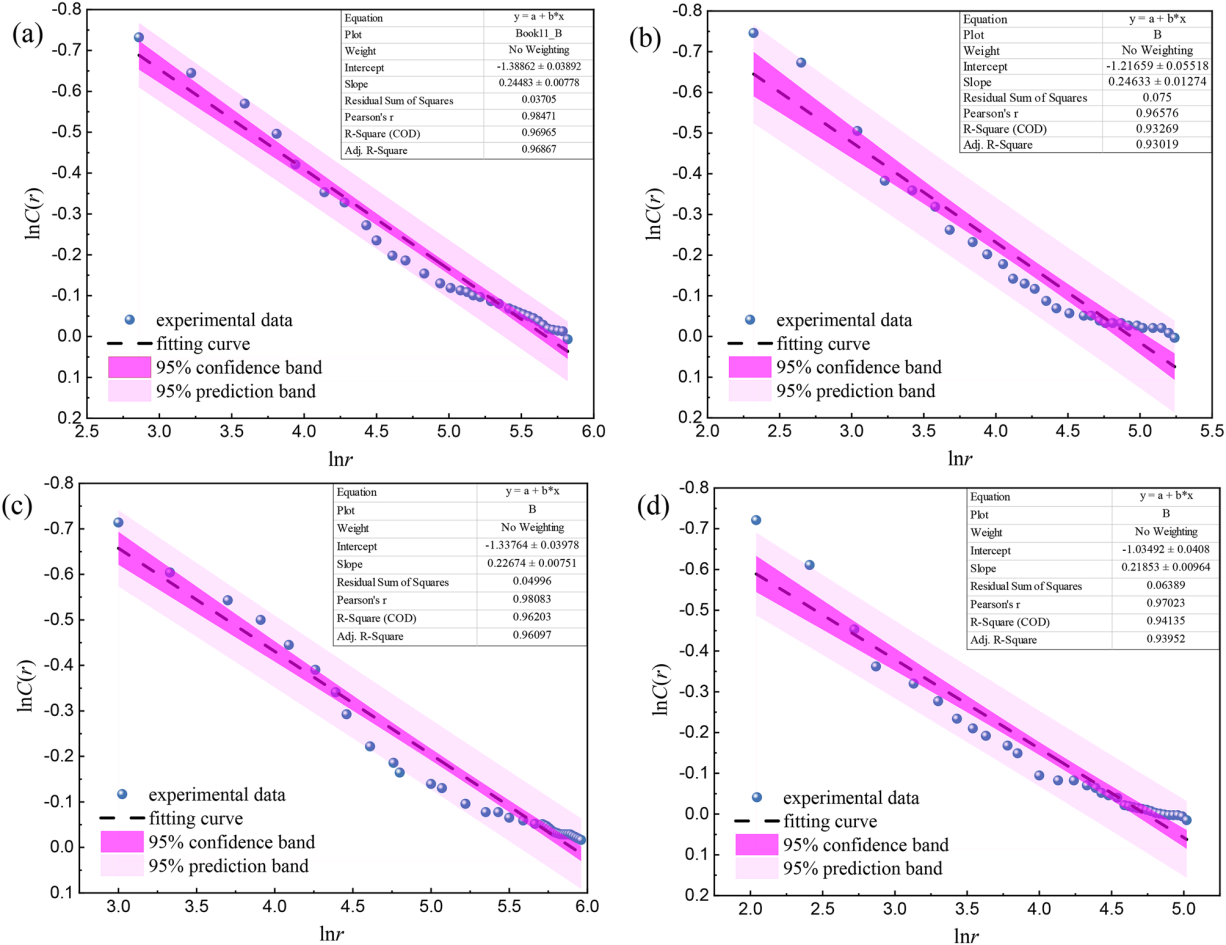
Curve of correlation dimension *D* of red beds soft rock with different water content: (**a**) natural state (**b**) 1 d (**c**) 3 d (**d**) 7 d.

Take 100 data from the whole loading process to calculate the correlation dimension *D*, and then obtain ln*C*(*r*) by Eq. (8), then the ln*C*(*r*)−ln*r* relationship curve is drawn in Fig. 15. The least square method is used to fit the experimental data. The correlation coefficients of different water content are all above 0.9, indicating that ringing counts of red beds soft rocks with different water content have significant monofractal characteristics, and the higher the correlation coefficient, the higher the self-similarity of AE ringing count time series. The correlation dimension *D* with different water content is between 0.21 and 0.25.

### AE multifractal characteristics of red beds soft rocks with different water content

In order to further analyze the fractal characteristics of AE time series, the box counting method is used to calculate the multifractal characteristics of AE time series^37,38^. Suppose the time series is {*x*_*i*_} divided into *N* subsets of length *ε*, then the probability distribution for each subset {*P*_*i*_(*ε*)} can be expressed by Eqs. (9)–(13):

9 $$ X_{q} \left( \varepsilon \right) = \sum {P_{i} \left( \varepsilon \right)^{q} } \sim \varepsilon^{\tau \left( q \right)} $$

10 $$ \tau \left( q \right) = \mathop {\lim }\limits_{\varepsilon \to 0} \frac{{\ln X_{q} \left( \varepsilon \right)}}{\ln \varepsilon } $$

11 $$ D_{q} = \left\{ {\begin{array}{*{20}l} {\frac{1}{q - 1}\mathop {\lim }\limits_{\delta \to 0} \frac{{\ln \sum {p_{i}^{q} \left( \delta \right)} }}{\ln \delta }} \hfill & {\left( {q \ne 1} \right)} \hfill \\ {\mathop {\lim }\limits_{\delta \to 0} \frac{{\sum {p_{i} \left( \delta \right)\ln p_{i} \left( \delta \right)} }}{\ln \delta }} \hfill & {\left( {q \ne 1} \right)} \hfill \\ \end{array} } \right. $$

12 $$ \alpha = \frac{{{\text{d}}\left( {\tau \left( q \right)} \right)}}{{{\text{d}}q}} = \frac{{\text{d}}}{{{\text{d}}q}}\left( {\mathop {\lim }\limits_{\varepsilon \to 0} \frac{{\ln X_{q} \left( \varepsilon \right)}}{\ln \varepsilon }} \right) $$

13 $$ f\left( \alpha \right) = \alpha q - \tau \left( q \right) $$

where *X*_*q*_(*ε*) is the partition function, also known as the statistical moment. *τ*(*q*) is a multifractal characteristic function, also known as the characteristic index. *q* is the weight, and the value range is (− ∞, + ∞), which represents the degree of non-uniformity of multifractals. When *q* is large, it means that large fluctuations dominate the entire time series. When *q* is small, it means that small fluctuations dominate the entire time series. *D*(*q*) is the generalized fractal dimension, and the greater the deviation between *D*(*q*) and 1, the greater the volatility of the data and the stronger the multifractal characteristics. *α* is a characteristic index, reflecting the degree of non-uniformity of the probability subset. *f*(*α*) is the fractal dimension of *α* subset, representing the frequency of the AE signal subset with *α* singularity during loading.

**Figure 16:**
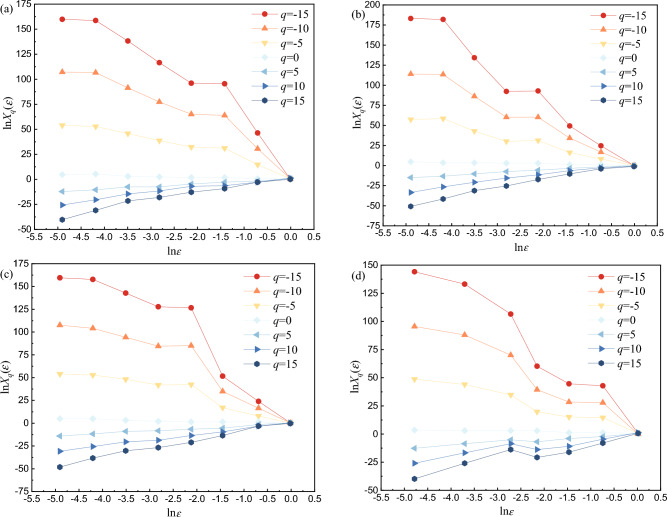
Distribution function of AE ringing count of red beds soft rock with different water content: (**a**) natural state (**b**) 1 d (**c**) 3 d (**d**) 7 d.

Based on Eqs. (5) to (9), Matlab mathematical analysis software is used to solve the distribution function curves of AE events of red beds soft rock with different water content are shown in Fig. 16. The *q* ranges from − 15 to 15 and the interval is 5. ln*X*_*q*_(*ε*) and ln(*ε*) have a linear relationship, especially when *q* < 0, ln*X*_*q*_(*ε*) and ln(*ε*) have a significant linear relationship. It shows that the AE ringing count of red beds soft rock with different water content has multifractal characteristics.

**Figure 17:**
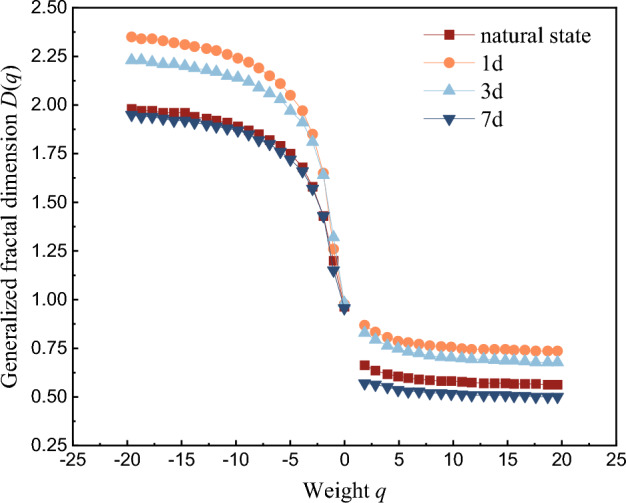
Multifractal dimension evolution curve of AE ringing count.

**Figure 18:**
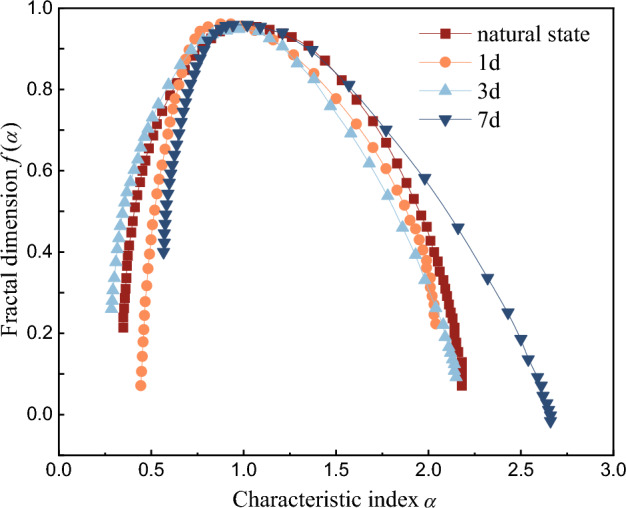
Multifractal spectrum curve of AE ringing count.

The multifractal dimension curves in Fig. 17 and multifractal curves in Fig. 18 of AE ringing count of red beds soft rock with different water content are also calculated using Matlab. It can be seen from Fig. 17, with the gradual increase of weight *q*, the generalized fractal dimension *D*(*q*) shows a gradually decreasing trend. Which indicates that AE ringing counts of red beds soft rocks with different water content during uniaxial fractional loading have multifractal characteristics. Moreover, *D*(*q*) ≠ 1 indicates that AE ringing counts show uneven distribution. When *q* < 0, under the same *q*, *D*(*q*) presents a trend of first increasing and then decreasing, and the cut-off point is 1 d. When *q* > 0, the variation trend of *D*(*q*) is roughly the same as that when *q* < 0, indicating that the water content has a significant influence on the crack propagation law of red beds soft rock.

It can be seen from Fig. 18, the multifractal spectrum variation of AE ringing counts with different water content is roughly the same, showing an inverted "U" shape, which indicates that the crack development process is similar. However, due to the different multifractal parameters, the micro-crack development characteristics with different water content are different.

According to the Matlab calculation results, the obtained multifractal spectrum parameters are shown in Table 2. Δ*α* = *α*_max_ − *α*_min_ represents the spectral width of the multifractal spectrum, which can represent the local characteristics of AE ringing count in the fractal structure. The corresponding subsets of *α*_max_ and *α*_min_ represent the strong signal and the weak signal in the AE time series respectively. The larger Δ*α* is, the more severe the signal fluctuation is, and the stronger the uneven distribution of AE ringing is. Δ*α*_L_ = *α*(*f*_max_) − *α*_min_ is the left width of the multifractal spectrum, indicating the proportion of strong signals. Δ*α*_R_ = *α*_max_ − *α*(*f*_max_) is the right width of the multifractal spectrum, indicating the weak signal proportion. Δ*f* = *f*(*α*_max_) − *f*(*α*_min_) represents the frequency relationship between different signals of different sizes, where Δ*f* < 0 indicates that weak signals dominate and are related to cracks, and Δ*f* > 0 indicates that strong signals dominate and are related to crack closure and propagation.

**Table 2:** Multifractal spectrum parameters of AE events.

Multifractal spectrum parameters	Immersion time			
	Natural state	1 d	3 d	7 d
*∆a*	1.68	1.92	1.85	1.70
*∆a*_*L*_	0.61	0.35	0.45	0.60
*∆a*_*R*_	1.02	1.54	1.34	1.07
*∆f*	− 0.15	− 0.44	− 0.02	0.16
*∆σ* (MPa)	49.01	46.54	44.32	43.01

The Δ*α* value of the red beds soft rock changes with the increase of water content, which indicates that the internal structure changes under the influence of water. From the natural state to immersed 1 d, the value of ∆*α* gradually increases, and the distribution uniformity of AE events gradually decreases. From 1 to 7 d, the ∆*α* value decreases gradually, the AE event distribution uniformity increases gradually, and the damage complexity decreases gradually. When saturation is not reached, ∆*f* < 0, indicating that the number of cracks in the specimen is small. When saturation is reached, ∆*f* > 0, indicating that a large number of cracks occur inside the specimen at this time, and macro fracture are formed. When the characteristic index *α* decreases, the compressive strength also decreases, which further indicates that there is a positive correlation between the multifractal spectrum parameters of AE ringing count and the mechanical properties.

## Discussion

From the above analysis, the AE ringing parameters of the water-bearing red beds soft rock during the failure process of uniaxial fractional loading have significant fractal characteristics, which can accurately describe the development of the internal joints and fissures of the rock. The increase of correlation dimension *D* indicates that the rock damage is gradually increasing, and when the correlation dimension *D* increases to a certain value, it begins to decrease, indicating that cracks in the rock begin to communicate and form macroscopic cracks. Water plays an important role in the rock failure process, and then reduce the correlation dimension *D*. Immersion time from 0 to 7 d, ∆*α* value increases first and then decreases. When the value ∆*α* increases, the distribution uniformity of AE events decreases. When the specimen does not reach saturation, ∆*f* < 0, which indicates that the number of cracks is small. When saturation is reached, ∆*f* > 0 indicates that a large number of cracks occur inside the specimen, and macro cracks are formed.

**Figure 19:**
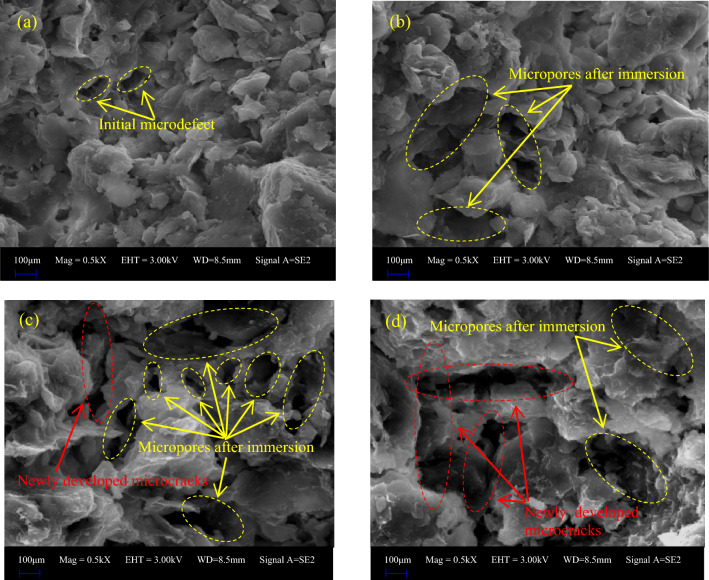
SEM images of red beds soft rock with different water content: (**a**) natural state (**b**) 1 d (**c**) 3 d (**d**) 7 d.

In order to further reveal the influence of water on the physical characteristics of red beds soft rock, SEM are carried out on the damaged specimens after different immersion times. Figure 19 shows the microstructure of red beds soft rock under different water contents. In the natural state, the interior of red beds soft rock is relatively dense, the particles are closely arranged, and the micro-cracks are few. With the prolongation of immersion time, the micro-pore structure gradually loosened, the intergranular pores gradually increased, the micro-cracks gradually expanded, and the local intergranular cementation was destroyed, squeezed and relatively slipped, resulting in the formation of solution micro-pores.

Through the above analysis, it was found that there is a certain positive correlation between the AE characteristics and microstructure of red beds soft rocks with different water contents. When the specimen is in the natural state, the AE ringing signal is strong, and the internal structure is mainly granular structure, strip structure and sheet structure. The main contact mode of internal minerals is relatively dense face-to-face or face-to-face edge contact, with fewer initial internal defects. With the gradual increase of water content, AE ringing signal gradually weakened, and the internal structure gradually changed into flocculent structure and flap structure. The contact mode of internal minerals becomes relatively loose edge-to-edge contact, and the micro-defects caused by immersion gradually increase, and through to form micro-cracks. The change in AE ringing signal results in different microstructures inside the red beds soft rock. For specimens with weak AE ringing signals, the bonding force between particles is smaller and the pores are larger. This microstructure will cause more refraction and reflection of the AE ringing signal when passing through a fixed length specimen, resulting in a longer time and weaker signal. Specimens with low adhesion between particles and high porosity have lower density and poorer properties, resulting in lower strength. Therefore, the AE ringing signal of red beds soft rock with different water content has a certain positive correlation with microstructure.

## Conclusion

1. With the gradual increase of water content, the AE ringing count before the yield stage gradually decreases, the corresponding cumulative ringing count at the same time gradually decreases, the cumulative ringing curve gradually slows down, the time of internal cracks is advanced, the cumulative ringing curve step is gradually significant, the AE signal amplitude is gradually weakened, and the bandwidth of each frequency band is gradually reduced. The failure of red beds soft rock with different water content is dominated by shear crack, and with the gradual increase of water content, the proportion of shear crack increases gradually, and the AE *b* value decreases gradually.
2. With the gradual increase of the relative peak strength, the correlation dimension *D* of red beds soft rock with different water content increases first and then decreases. At 80% of the relative peak strength, the correlation dimension *D* reaches its maximum value, and then drops sharply until it is maintained at a relatively low level, and the correlation dimension *D* gradually decreases with the water content. The fitting correlation coefficients of different water content (ln*C*(*r*) and ln*r*) are all above 0.9, indicating that the AE ringing count has fractal characteristics, and the higher the correlation coefficient, the higher the self-similarity of AE ringing count sequence.
3. When *q* < 0, ln*X*_*q*_(*ε*) has a good linear relationship with ln(*ε*). As the weight *q* gradually increases, the generalized fractal dimension *D*(*q*) gradually decreases. When *q* ≠ 0, under the condition of the same *q* value, *D*(q) increases first and then decreases. The multifractal spectrum of AE ringing count of red beds soft rock with different water content is inverted “U” shape. With the gradual increase of water content, Δ*α* value of changes, from the natural state to immerse 1 d, Δ*α* value gradually increases, from immersing 1–7 d, Δ*α* value gradually decreases. When saturation is not reached, ∆*f* < 0, indicating that the number of cracks is small. When saturation is reached, ∆*f* > 0, indicating that a large number of cracks occur inside the specimen, and macro cracks are formed.

## Data Availability

All data, models, and code generated or used during the study appear in the submitted article.
